# Clinical and Brain Morphometry Predictors of Deep Brain Stimulation Outcome in Parkinson’s Disease

**DOI:** 10.1007/s10548-024-01054-2

**Published:** 2024-04-25

**Authors:** Maija Koivu, Aleksi J. Sihvonen, Johanna Eerola-Rautio, K. Amande M. Pauls, Julio Resendiz-Nieves, Nuutti Vartiainen, Riku Kivisaari, Filip Scheperjans, Eero Pekkonen

**Affiliations:** 1 Department of Neurology, Helsinki University Hospital and Department of Clinical Neurosciences (Neurology), University of Helsinki, Finland, Helsinki, Finland; 2 Cognitive Brain Research Unit, Department of Psychology and Logopedics, Faculty of Medicine, University of Helsinki, Helsinki, Finland; 3 Department of Neurosurgery, Helsinki University Hospital, Helsinki, Finland

**Keywords:** Parkinson’s disease, Directional deep brain stimulation, Brain morphometry, Deep brain stimulation, Subthalamic nucleus

## Abstract

Subthalamic deep brain stimulation (STN-DBS) is known to improve motor function in advanced Parkinson’s disease (PD) and to enable a reduction of anti-parkinsonian medication. While the levodopa challenge test and disease duration are considered good predictors of STN-DBS outcome, other clinical and neuroanatomical predictors are less established. This study aimed to evaluate, in addition to clinical predictors, the effect of patients’ individual brain topography on DBS outcome. The medical records of 35 PD patients were used to analyze DBS outcomes measured with the following scales: Part III of the Unified Parkinson’s Disease Rating Scale (UPDRS-III) off medication at baseline, and at 6-months during medication off and stimulation on, use of anti-parkinsonian medication (LED), Abnormal Involuntary Movement Scale (AIMS) and Non-Motor Symptoms Questionnaire (NMS-Quest). Furthermore, preoperative brain MRI images were utilized to analyze the brain morphology in relation to STN-DBS outcome. With STN-DBS, a 44% reduction in the UPDRS-III score and a 43% decrease in the LED were observed (*p*<0.001). Dyskinesia and non-motor symptoms decreased significantly [median reductions of 78,6% (IQR 45,5%) and 18,4% (IQR 32,2%) respectively, *p*=0.001 – 0.047]. Along with the levodopa challenge test, patients’ age correlated with the observed DBS outcome measured as UPDRS-III improvement (*ρ=* -0.466 – -0.521, *p*<0.005). Patients with greater LED decline had lower grey matter volumes in left superior medial frontal gyrus, in supplementary motor area and cingulum bilaterally. Additionally, patients with greater UPDRS-III score improvement had lower grey matter volume in similar grey matter areas. These findings remained significant when adjusted for sex, age, baseline LED and UPDRS scores respectively and for total intracranial volume (*p*=0.0041- 0.001). However, only the LED decrease finding remained significant when the analyses were further controlled for stimulation amplitude. It appears that along with the clinical predictors of STN-DBS outcome, individual patient topographic differences may influence DBS outcome. Clinical Trial Registration Number: NCT06095245, registration date October 23, 2023, retrospectively registered

## Introduction

Subthalamic deep brain stimulation (STN-DBS) is an evidence-based treatment option for advanced Parkinson’s disease (PD) (Deuschl et al. 2006, Mansouri et al. 2018). DBS treatment usually alleviates cardinal Parkinsonian motor symptoms, such as rigidity, tremor, and bradykinesia (Krack et al. 2003, Deuschl, Paschen et al. 2013).

Even after careful patient selection with well-established guidelines (Bronstein et al. 2011, Hartmann et al. 2019, Deuschl, Antonini et al. 2022), DBS response may vary among PD patients. A levodopa challenge test is considered to be a good predictor of DBS response (Charles et al. 2002, Lang et al. 2006, Lachenmayer et al. 2021, Lin et al. 2022). More than 33% improvement in Unified Parkinson’s Disease Rating Scale part III (UPDRS-III) score during the ON phase of the levodopa challenge test is required for STN-DBS treatment (Charles et al. 2002, Lang et al. 2006, Lachenmayer et al. 2021, Lin et al. 2022). Several studies have suggested that younger age may be a predictor of DBS response and quality of life after DBS operation (Charles et al. 2002, Lang et al. 2006, Groiss et al. 2009, Hartmann et al. 2019, Deuschl et al. 2022). In other studies, the relationship between aging and DBS response has not been confirmed (Geraedts et al. 2020, Lin et al. 2022). Additionally, the disease duration prior to the initiation of DBS treatment and fewer levodopa-resistant symptoms may be predictors of DBS response (Lachenmayer et al. 2021). On neuroimaging, vascular changes have been recognized as clinical predictors of poorer long-term DBS outcome (Cavallieri et al. 2021).

Versatile theories on the actions of DBS on Parkinsonian symptoms have been proposed. DBS may affect neuronal firing rates locally (Lozano and Lipsman 2013, Chiken and Nambu 2016) but may also modulate more remote connected brain regions (McIntyre and Hahn 2010, Lozano and Lipsman 2013, Horn et al. 2017, Johnson et al. 2020). This connectivity may predict DBS outcome in PD patients (Horn et al. 2017). An individual patient’s brain topographic features may also have an impact on DBS response (Frizon et al. 2020, Chen et al. 2022, Jergas et al. 2023). However, to date, reliable topographic predictors have not been identified. While some studies suggest that cortical thickness, especially in the motor and supplementary motor cortex, could predict motor outcome in DBS, other studies have provided contradictory evidence (Wang et al. 2022). Additionally, the volumes of various brain regions have been linked to DBS response, but consistent findings have not been reported (Younce et al. 2019, Yim et al. 2020). The aim of this study was to evaluate possible clinical patient features affecting DBS response and to investigate whether patients’ individual cortical volumes have an impact on DBS response.

## Materials and Methods

### Clinical Data

The medical records of 48 consecutive PD patients treated with STN-DBS at Helsinki University Hospital (HUS) were reviewed. MRI imaging was performed on 35 patients with the same MRI machine (3T Magnetom Skyra, Siemens Healthineers, Erlangen, Germany), and these patients were selected for this retrospective study. Staging of advanced PD in all patients had been determined by a treating movement disorder specialist according to Delphi criteria (Antonini et al. 2018). All patients had undergone a routine DBS screening (including a thorough neuropsychological examination, the levodopa challenge test and brain MRI scan without observed significant vascular changes). All patients received a directional DBS system (Abbott Infinity DBS™, Abbott Neuromodulation, Austin, TX, USA). The patients had completed a full DBS programming routine with programming visits at 1, 1.5, 3 and 6 postoperative months as described earlier (Koivu et al. 2022). Since the MRI imaging protocol for DBS was revised at HUS in 2019, only patients operated on between 2020 – 2022 were selected for this study.

The following clinical data were collected from the DBS screening visit (baseline) and the in-hospital six-month programming visit at HUS: UPDRS-III in the medication off state and in the medication ON state (during the levodopa challenge test) and in the medication off, stimulation on state (at the six-month visit), Abnormal Involuntary Movements Scale (AIMS), Parkinson’s Disease Questionnaire 39 (PDQ-39), Mini Mental State Examination (MMSE), Non-Motor Symptoms Questionnaire (NMS-Quest), and Beck Depression Inventory (BDI). Additionally, data on the use of antiparkinsonian drugs and other comorbidities were obtained. The levodopa equivalent dose (LED) was calculated as previously proposed (Tomlinson et al. 2010, Schade et al. 2020). The DBS programming settings (amplitudes, pulse width, frequency, impedance) as well as the stimulation mode and possible alterations in electrode activation during the six-month follow-up were collected. The research permit was approved by the medical director responsible for the academic research at HUS without additional approval from the ethics committee according to Finnish laws.

### MRI Data Acquisition

All neuroimaging data were collected at the Department of Radiology. High-resolution magnetization-prepared rapid acquisition gradient-recalled T1 images were obtained (TR = 2000 ms, TE = 2.74 ms, voxel size = 1 × 1 × 1). A neuroradiologist checked the MRI images for incidental findings.

### Voxel-based Morphometry

Morphometric analysis was carried out using Statistical Parametric Mapping (SPM12, Wellcome Department of Cognitive Neurology, UCL) in MATLAB 9.10.0 (The MathWorks, Inc., Natick, MA, USA; version R2021a). First, the individual T1 images were reoriented using the anterior commissure as the origin. The new segmentation algorithm with default parameters, except for the affine regularization set to the International Consortium for Brain Mapping (ICBM) template for the brains of European participants, was subsequently applied to the T1 images, segmenting them precisely into grey matter, white matter, and cerebrospinal fluid probability maps. The tissue probability maps were then normalized to the Montreal Neurological Institute (MNI) space using the Diffeomorphic Anatomical Registration Through Exponentiated Lie Algebra (DARTEL) registration process implemented in SPM12. During the normalization process, the data were resampled to a 1.5 × 1.5 × 1.5 mm^3^ voxel size and modulated, allowing evaluation of regional volumetric differences. Finally, grey matter and white matter maps were smoothed with an isotropic Gaussian kernel of 8 mm full width at half maximum (FMWH). During each step, the images were visually checked for potential segmentation and registration errors. The total intracranial volume for each patient was calculated by combining the grey matter, white matter, and cerebrospinal fluid images generated during the segmentation.

### Statistical Analyses

All the statistical analyses were performed with IBM SPSS Statistics, version 27 (IBM Corporation, Armonk, New York, USA). The data are presented as medians with interquartile ranges (IQRs), and p values less than 0.05 were considered significant. Clinical data analyses were conducted using the Wilcoxon signed rank test and Mann‒Whitney test considering the small patient cohort, and for correlations, the Spearman correlation test or Pearson two-tailed correlation when applicable, contingency coefficient, and linear regression were used for DBS outcome analysis.

In the voxel-based morphometry analysis, two linear regression models evaluating the relationship between pre-DBS regional grey matter volume and changes (6 months > preoperative) in i) LED and ii) UPDRS-III scores were calculated using SPM12. The results were thresholded using the “Threshold and transform spmT-maps” function in the CAT12 toolbox at a default cluster-forming threshold (uncorrected p < 0.001) and a familywise error rate (FWE) corrected p < 0.05 at the cluster level (alpha-level) and corrected for non-isotropic smoothness (Hayasaka et al. 2004). All voxel-based morphometry analyses were adjusted for age, sex, and total intracranial volume (Barnes et al. 2010) as well as for baseline LED and UPDRS-III scores (Hope et al. 2019). Neuroanatomical regions were identified using the Automated Anatomical Labeling Atlas (Tzourio-Mazoyer et al. 2002) included in the xjView toolbox (http://www.alivelearn.net/xjview/).

## Results

### Clinical Data

The baseline demographic data of the 35 PD patients are presented in Table 1.

**Table 1:** The preoperative demographics of the patients

Median (IQR)	
Age (years)	60,0 (12,0)
Parkinson’s disease duration (years)	10,0 (2,0)
Duration of advanced Parkinson’s diseaseˣ (years)	2,0 (3,0)
Sex (male/female)	19/16
Comorbidities and other significant medication	18 patients (51,4%) had no other comorbidities. 8 patients (22,8%) had a stable CAD. 3 patients (8,6%) had diabetes mellitus, type II. 3 patients (8,6%) had had depression and used antidepressant.^¶^ 2 patients (5,7%) were in remission of previously treated malignancy. 1 patient (2,9%) had atrial fibrillation with anticoagulation therapy.
The indication for DBS treatment	Severe and trouble-some motor fluctuations with 30 patients (85,7%). Severe dyskinesia with 4 patients (11,4%). Severe levodopa responsive tremor along with other milder motor symptoms with 1 patient (2,9%).

^ˣ^As reported by the patients, when especially asked and evaluated with Delphi criteria by the treating movement disorder specialist (Antonini et al. 2018), ^¶^none of these patients showed signs of clinical depression during the neuropsychological evaluation. *CAD* coronary artery disease

During the levodopa challenge test at the DBS screening visit, a median decrease of 23,0 points (IQR 10,0 points) in the UPDRS-III score was observed. This corresponds to a median improvement of 65% (IQR 20,5%) (Table 2). After 6 months of DBS treatment, the median UPDRS-III score was reduced by 15,0 points (IQR 15,0 points) when assessed during medication off, stimulation on, corresponding to a 43,9% (IQR 25,6%) decrease in the UPDRS-III score (Table 2). In addition, non-motor symptoms, dyskinesia (as evaluated with the AIMS) and motor symptoms (as evaluated with the H&Y scale in medication off condition) also improved significantly with DBS treatment. The postoperative decrease in LED was also significant, with a median reduction of 43,0% (IQR 33,1%) (*p*<0.001).

**Table 2:** Clinical rating scores at baseline and after 6 months of DBS treatment

	Baseline		Six months		p values
	Median score	IQR	Median score	IQR	
UPDRS-III, med off (points)	36,0	9,0	21,0 (stimulation on)	10,0	*p*<0.001
UPDRS-III after the levodopa challenge (points)	12,0	6,0	*not performed at six-month visit*		
LED (milligrams)	1251,0	385,0	771,5	579,0	*p*<0.001
AIMS (points)	7,5	9,0	2,0	3,0	*p*<0.001
H&Y, med off (stages)	3,0	0,5	2,0 (stimulation on)	0,5	*p*<0.001
NMS-Quest (points)	10,0	10,0	8,0	8,0	*p*=0.047
PDQ-39 SI (points)	31,0	30,2	24,3	27,0	*p*=0.201
BDI (points)	6,0	6,0	6,0	8,0	*p*=0.483
MMSE (points)	29,0	2,0	29,0	3,0	*p*=0.216

*UPDRS-III* Unified Parkinson’s Disease Rating Scale Part III, *med off* medication off condition, *LED* levodopa equivalent dose, *AIMS* Abnormal Involuntary Movement Scale, *H&Y* Hoehn et Yarh scale, *NMS-Quest* Non-motor Symptoms Questionnaire, *PDQ-39 SI* Parkinson’s Disease Questionnaire 39 Summary Index, *BDI* Beck Depression Inventory, *MMSE* Mini-Mental State Examination

Patients’ age correlated with the duration of PD (*ρ=*0.505, *p*=0.002) but not with the duration of the advanced stage of PD (*ρ=*0.040, *p*=0.834). Furthermore, age did not correlate with baseline UPDRS-III score or LED, sex, or PDQ-39 SI score, nor did the aforementioned variables correlate with each other (*ρ=*-0.294 – 0.443, *p*=0.086 – 0.945). Age correlated with longitudinal UPDRS-III score improvement with STN-DBS and with observed improvement in the levodopa challenge test score (*ρ=*-0.466 – -0.521, *p*<0.005) but not with longitudinal LED decrease (*ρ=*-0.072, *p*=0.682). Younger patients had greater improvements in the UPDRS-III scores with STN-DBS and greater improvements in motor symptoms the levodopa challenge test. Age appeared to predict UPDRS-III score improvement with DBS stimulation (β=-0.595, *p*<0.001) but did not predict LED decrease after DBS therapy (β=-0.070, *p*=0.688).

Motor improvement in the levodopa challenge test correlated with longitudinal UPDRS-III score changes (*ρ=*0.478, *p*=0.004) but not with disease duration, sex, or the reported duration of advanced PD (*ρ=*0,024 – -0.286, *p*=0.101 –0.898). The longitudinal UPDRS-III score improvement did not correlate with the baseline LED, disease duration or reported duration of advanced PD (*ρ=*-0.289 – 0.014, *p*=0.097-0.936) or with the baseline PDQSI or NMS-Quest scores (*ρ=*-0.170– 0.244, *p*=0.097 – 0.871). Disease duration and self-reported duration of advanced PD were not associated with longitudinal UPDRS-III score changes (β=-0.299–0.023, *p*=0.165–0.913).

### DBS data

After six months, directional stimulation was used in 87,1% of the dDBS leads (in 61 out of 70 dDBS leads); see Table 3. With 27 patients, directional single-segment stimulation (SSA) was used bilaterally, and with others, SSA was activated in one of the dDBS leads, as was omnipolar and/or two-segment activation in the other leads. Only two patients received omnidirectional stimulation bilaterally after directionality testing.

**Table 3:** The median DBS settings after six months of follow-up

Median (IQR)	
Stimulation type	1 active segment^¶^ in 58/70 (82,9%) electrode contacts 2 active segments in 3/70 (4,3%) 3 active segments^ˣ^ in 9/70 (12,8%)
Amplitude (mA)	2,2 (0,93)
Pulse width (μs)	60,0 (3,0)
Frequency (Hz)	130,0 (0,0)
Impedance (Ω)	1681,0 (647,0)

^¶^Single-segment activation (SSA), ˣ=omnipolar stimulation, *mA* milliamperes, *μs* microseconds, *Hz* hertz, *Ω* ohms

There were no operation-related complications (infection or intracranial bleeding) or severe stimulation-related adverse effects (e.g., dysarthria or depression). One patient had deep vein thrombosis postoperatively treated with a regimen of transient anticoagulation therapy.

### Morphometric analysis

First, the relationships between longitudinal changes in LED and UPDRS-III scores and global brain measurements (i.e., grey matter, white matter, cerebrospinal fluid and total brain volume) as defined by the percentage out of total intracranial volume (e.g.,$$\frac{grey matter volume}{total intracranial volume}$$) were evaluated with Pearson correlations (two-tailed). No statistically significant associations were found between the global brain measures and LED change (grey matter r=-0.255, *p*=0.140; white matter r=0.290, *p*=0.082; cerebrospinal fluid r=-0.103, *p*=0.555; and total brain volume r=0.103, *p*=0.555) or UPDRS-III score change (grey matter r=0.032, *p*=0.853; white matter r=0.064, *p*=0.717; cerebrospinal fluid r=-0.237, *p*=0.171; and total brain volume r=0.237, *p*=0.171).

**Fig. 1:**
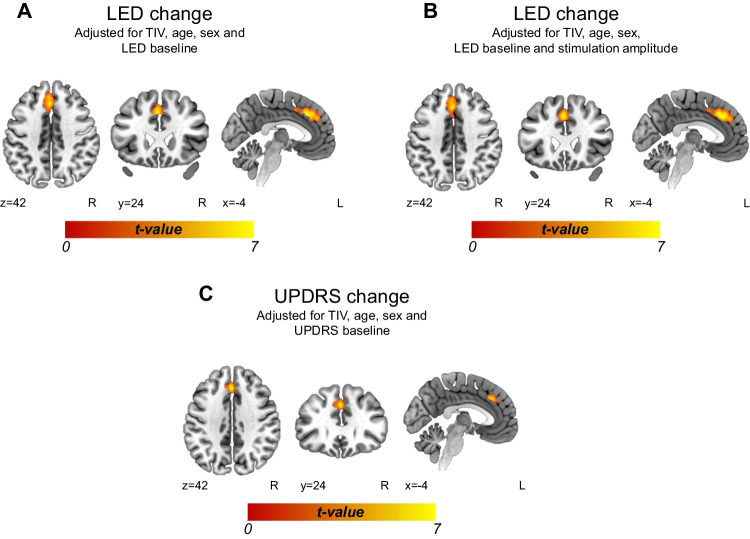
Morphometric results showing grey matter volume associations with longitudinal changes in LED and UPDRS-III scores. (**A**) and (**B**) lower baseline grey matter volume predicting greater longitudinal decrease in LED, and (**C**) lower baseline grey matter volume predicting greater longitudinal decrease in the UPDRS-III score. N = 35. The results are reported using MNI coordinates at the cluster-forming threshold (uncorrected p < 0.001) and a FWE-corrected p < 0.05 at the cluster level (alpha level) and corrected for non-isotropic smoothness. L = Left, R = Right, TIV = Total intracranial volume

In the whole-brain voxel-based morphometry VBM analyses adjusted for total intracranial volume, age, sex and baseline LED, a greater longitudinal decrease in LED was associated with lower grey matter volume in one cluster comprising the left superior medial frontal gyrus as well as the supplementary motor area and cingulum bilaterally (Fig. 1A, Table 4). The finding remained relatively unchanged when the analysis was further adjusted for the stimulation amplitude used (Fig. 1B). The duration of PD or the duration of advanced PD did not show correlations with aforementioned grey matter clusters observed with LED decrease (disease duration r=0.014, *p*=0.938; advanced PD r=0.004, *p*=0.983).

**Table 4:** Baseline grey matter volume clusters predicting longitudinal changes in LED and UPRDS-III scores

Condition	Area name	Coordinates	Cluster size	t value	P_FWE_-value
LED	Left Frontal Superior Gyrus (Medial) (BA 9)	-9 33 32	1537	5.38	<0.001
	Left Cingulum Anterior (BA 32)	-4 33 30			
	Right Cingulum Anterior and Mid (BA 32)	4 36 26			
	Left Supplementary Motor Area (BA 6)	-3 11 46			
	Right Supplementary Motor Area (BA 6)	4 12 45			
UPDRS score	Left Frontal Superior Gyrus (Medial) (BA 8, 9)	-2 30 36	415	5.23	0.041
	Left Cingulum Anterior (BA 32)	-2 37 28			
	Right Cingulum Anterior and Mid (BA 32)	4 44 21			
	Right Frontal Superior Gyrus (Medial) (BA 6)	4 27 40			

*BA* Brodmann area, *LED* levodopa equivalent dose, *UPDRS* Unified Parkinson Disease Rating Scale Part III

**Fig. 2:**
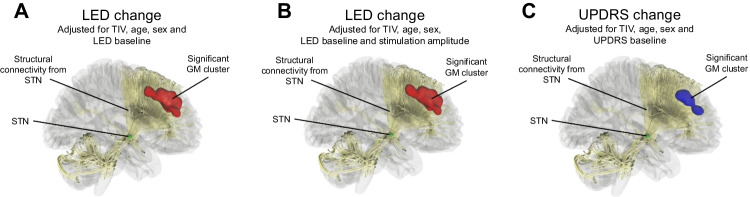
Visualization of the significant morphometric findings in relation to the structural connectivity of the subthalamic nucleus. (**A**) and (**B**) lower baseline grey matter volume predicting greater longitudinal decrease in LED, and (**C**) lower baseline grey matter volume predicting greater longitudinal decrease in UPDRS score. GM = Grey matter, STN = subthalamic nucleus, TIV = total intracranial volume

A greater decrease in the UPDRS-III score was associated with a lower grey matter volume in a similar cluster comprising the superior medial frontal gyrus and the cingulum bilaterally (Fig. 1C, Table 4) when the analysis was adjusted for total intracranial volume, age, sex, and baseline UPDRS score. However, when the analysis was further adjusted for the stimulation current amplitude, no significant findings were observed. Observed grey matter findings associated with UPDRS-III improvements did not correlate with the disease duration nor with the duration of advanced PD (disease duration r=0.040, *p*=0.822; advanced PD r=0.018, *p*=0.926). Figure 2 shows the structural connectivity of the STN in relation to the significant grey matter findings.

## Discussion

Using clinical variables and grey matter morphometric MRI analyses, this study aimed to evaluate predictors of STN-DBS outcome in advanced PD patients. Our findings indicate that both age and the levodopa challenge test score significantly predict STN-DBS outcome, measured as UPDRS-III score improvement. Furthermore, lower grey matter volume in the left superior frontal/supplementary motor areas (SMA) and cingulum predicted greater longitudinal UPDRS-III improvement and LED decrease in this patient cohort. The disease duration or the duration of advanced stage of PD did not correlate with the morphometric findings in this study. Overall, the previous morphometric studies on DBS effect have been heterogeneous, presenting various brain regions’ role in connectivity with STN in the DBS outcome. Previous studies have shown that multiple brain regions are both positively and negatively correlated with postsurgical outcome (Wang et al. 2022). This can be explained to some extent by the various research methods used. In some studies, the thickness of the motor cortex predicted motor outcome after DBS operation, and in others, these results could not be replicated (Wang et al. 2022). In a study by Muthuraman (Muthuraman et al. 2017), the thickness of the frontal cortex, principally in the paracentral area and superior frontal region, was found to be a predictor of motor improvement following STN-DBS treatment. In another study, a positive association between STN-DBS outcome and stimulated area’s connections to the SMA and the right precentral cortex was noted (Chen et al. 2022). Additionally, in the same study, a negative association between DBS outcome and connectivity to the right superior frontal, middle and inferior frontal cortices, anterior and middle cingulum and caudate was reported (Chen et al. 2022). Preoperative STN connectivity to the frontal and prefrontal cortex and cingulate gyrus has been correlated with postoperative outcomes (Koirala et al. 2018, Gonzalez-Escamilla et al. 2022), and long-term STN-DBS outcomes have been associated with connectivity to the SMA (Chen et al. 2022).

Posterior and middle STN have strongest connectivity to the primary motor cortex and SMA (Obeso et al. 2008, DiRisio et al. 2023), and the SMA activation is speculated to result from the activation of the fibers in the hyperdirect pathway (Accolla et al. 2016, Horn et al. 2017). In neuroimaging studies with ongoing subthalamic stimulation, increased cerebral blood flow has been noted in SMA, anterior cingulate and prefrontal cortex (Ceballos-Baumann et al. 1999, Sestini et al. 2005). In a study with functional MRI, an augmented interconnectedness in the motor cortices bilaterally and enhanced cerebello-thalamic-cortical network connectivity were observed with STN DBS treatment but not with oral levodopa treatment (Mueller et al. 2018). It has been suggested that broad frontal and premotor cortical thinning can be found in patients with PD compared to healthy controls (Ibarretxe-Bilbao et al. 2012, Pereira et al. 2014), and this phenomenon may have influenced results in this study. In addition to frontal cortical connections, studies have demonstrated associations with other brain regions and STN-DBS outcomes. A greater thickness of the left lateral occipital lobe has been reported to be associated with better UPDRS-III score improvement after STN-DBS (Frizon et al. 2020). This observation was noted in region of interest (ROI) analysis as well as in vertex-by-vertex analysis. In the same study, a thicker frontal and temporal cortex was found to be associated with UPDRS-III score improvements in ROI analysis but not in vertex-by-vertex analysis (Frizon et al. 2020). In a recent study, grey matter atrophy in the medial prefrontal cortex, cingulate gyrus, paracingulate gyrus and parietal lobe was related to a suboptimal response to DBS, although a significant association with brain atrophy and the levodopa response could not be detected (Jergas et al. 2023).

Some studies suggest that subcortical brain structures are linked to DBS outcome (Price et al. 2011, Younce et al. 2019, Yim, Kim et al. 2020). Smaller thalamus volumes and larger ventricle volumes have been proposed to be predictors of poor motor improvements after DBS surgery (Younce et al. 2019, Yim et al. 2020), although in another study, ventricle volumes lacked predictive value (Price et al. 2011). Moreover, previous studies lack consistency in the utilization of relevant covariates necessary for volumetric analyses (Wang et al. 2022). Furthermore, to the best of our knowledge, only one previous study has adjusted the analyses for baseline LED and UPDRS-III score (Younce et al. 2019), as recommended (Hope et al. 2019). However, to the best of our knowledge, not all of those previous studies controlled the stimulation amplitude. Taken together, the previous heterogeneous findings may be partly explained by the methodological and statistical variation as well as the lack of consistency in adjusting the analyses for confounding effects in the studies, although the demographics of the patients were relatively homogeneous.

In this study, the data were corrected for non-isotropic smoothness (Hayasaka et al. 2004) and all analyses were adjusted for age, sex, and total intracranial volume (Barnes et al. 2010) as well as for baseline levodopa equivalent daily dose and UPDRS-III scores (Hope et al. 2019). Greater declines in the UPDRS-III score were observed with lower grey matter volume in the superior medial frontal gyrus and cingulum bilaterally (see Fig. 1C); however, when these changes were further controlled by the stimulation current amplitude, no significant changes were observed. In some studies, higher voltages or amplitudes were needed for an optimal stimulation-induced improvement in those with thinner frontal cortex (Muthuraman et al. 2017, Koirala et al. 2018, Gonzalez-Escamilla et al. 2022). In the present study, with current stimulation, no relationship was observed between the required stimulation current for optimal motor control and the DBS response. In all the patients, the amplitudes used were rather moderate (median, 2.2 mA; IQR, 0.9 mA). Similarly, a longitudinal decrease in LED seemed to be associated with a decrease in grey matter volume in the left superior medial frontal gyrus as well as in the supplementary motor area and cingulum bilaterally (see Fig. 1A), even when controlling stimulation amplitude. These observations may be related to our small patient cohort, though its size is comparable to that of many previous morphometric studies (Muthuraman et al. 2017, Frizon et al. 2020). Therefore, additional studies are warranted to reproduce these results.

The clinical results of the six-month STN-DBS treatment in this study paralleled those of previous studies; approximately 44% improvement in the UPDRS-III score and a 43% decrease in the LED were observed, as was a significant reduction in dyskinesia. With STN-DBS, 43–51% improvements in UPDRS-III scores and 50–64% LED reductions have been reported (Bronstein et al. 2011, Dembek et al. 2017, Shao et al. 2020, Schnitzler et al. 2022). For 27 patients (77%), bilateral directional single-segment stimulation was used, only two patients preferred bilateral ring-mode stimulation, and all the stimulation parameters used resembled previous reports (Rammo et al. 2022, Schnitzler et al. 2022).

In the present study, age and the levodopa response in the levodopa challenge test predicted a significantly positive DBS response as assessed by the UPDRS-III score. However, age did not seem to influence the reduction in LED with DBS treatment. Earlier reports on the impact of aging on DBS response have been heterogeneous. In some studies, age has been shown to be negatively correlated with postoperative DBS responsiveness, as measured by the reduction in the UPDRS-III score (Charles et al. 2002, Russmann et al. 2004), and improved motor function has been noted with younger age and shorter disease duration (Welter et al. 2002, Hartmann et al. 2019). According to a recently published meta-analysis of STN- and GPi-DBS-treated PD patient cohorts, preoperative levodopa responsiveness predicted short-term (6- to 12-month) DBS responsiveness, whereas age did not have predictive value (Lin et al. 2022). Similar results for aging have been published previously (Kleiner-Fisman et al. 2006, Weaver et al. 2009, Hartmann et al. 2019, Geraedts et al. 2020, Lin et al. 2022).

This study has limitations due to its retrospective nature. The patient population was relatively small; therefore, an extensive statistical analysis of DBS outcomes could not be performed.

Another limitation to the study is that post-operational survey of the DBS lead location was not conducted. With these patients, all electrode contacts had been tested systematically with previously presented routine (Koivu et al. 2022). Each electrode contact had been tested initially in omnipolar stimulation mode during 1-month programming visit and then in directional mode at 1.5-month visit for determining the most optimal contact for stimulation. Patients had a notable clinical improvement (as measured with UPDRS-III scores) and a significant LED reduction. Based on that, it could be assumed that the active electrode was within or directly adjacent to the motor region of STN and its fibers connected to cortico-striato-thalamocortical motor loop. The methodology of this study is comparable to the methods used in previous reports (Muthuraman et al. 2017, Frizon et al. 2020). Some studies have shown that a DBS electrode’s connectivity profile depends on the shape, size and position of the volume of tissue activated (VTA) (Horn et al. 2017, Chen et al. 2022). Conversely, a study with insignificant correlation between the postoperative UPDRS-III and the position of the VTA has been published (Koirala et al. 2018). The method of calculating VTA also has some limitations, including being biased on assumptions about white matter fiber orientation and tissue homogeneity which are not always met (Koirala et al. 2018, Duffley et al. 2019).

In conclusion, individual patient morphometric properties, especially in cortical areas, may predict motor outcomes in STN-DBS in combination with clinical predictors and DBS settings.

## Data Availability

Data available upon a request.
